# Study Design and Quality of Reporting of Randomized Controlled Trials of Chronic Idiopathic or Autoimmune Urticaria: Review

**DOI:** 10.1371/journal.pone.0070717

**Published:** 2013-08-05

**Authors:** Elodie Le Fourn, Bruno Giraudeau, Olivier Chosidow, Marie-Sylvie Doutre, Gérard Lorette

**Affiliations:** 1 CHRU de Tours, Service de Dermatologie, Tours, France; 2 Université François-Rabelais de Tours, PRES Centre-Val de Loire Université, Tours, France; 3 CHRU de Tours, Tours, France; 4 INSERM Centre d’Investigation Clinique 202, Tours, France; 5 UPEC-Université Paris-Est Créteil Val de Marne, Créteil, France; 6 Hôpital Henri Mondor, Department of Dermatology, Créteil, France; 7 INSERM Centre d’Investigation Clinique 006, Créteil, France; 8 Hôpital Haut-Lévêque, Departement of Dermatology, Pessac, France; The University of Queensland, Australia

## Abstract

**Background:**

The recommended first-line therapy of chronic urticaria is second-generation antihistamines, but the modalities of treatment remains unclear. Numerous recommendations with heterogeneous conclusions have been published. We wondered whether such heterogeneous conclusions were linked to the quality of published studies and their reporting.

**Objective:**

To review the study design and quality of reporting of randomized control trials investigating pharmacological treatment of autoimmune or idiopathic chronic urticaria.

**Methodology/Principal Findings:**

MEDLINE and EMBASE were searched for pharmacological randomized controlled trials involving patients with chronic autoimmune or idiopathic urticaria, with the main outcome being treatment efficacy. Data were collected on general characteristics of the studies, internal validity, studied treatments, design of the trial, outcome measures and “spin” strategy in interpreting results. Spin was defined as use of specific reporting strategies to highlight that the experimental treatment is beneficial, despite statistically nonsignificant results. We evaluated 52 articles that met our criteria. Patients were reported as blinded in 42 articles (81%) and the outcome assessor was blinded in 37 (71%). A placebo was the only comparator in 13 (25%) studies. The study duration was <8 weeks in 39 articles (75%), with no follow-up after discontinuation of treatment in 37 (71%). In 4 articles (8%), blinding was clear because they described blinding of the outcome assessor, the treatment was not recognizable (identical or double-dummy) or had no major secondary effects, and computed randomization was centralized. The primary outcome was specified in 33 articles (63%) and was a score in 31. In total, 15 different scores were used. A spin strategy was used for 10 of 12 studies with a nonsignificant primary outcome.

**Conclusion:**

For establishing guidelines in treatment of chronic urticaria, studies should focus on choosing clinically relevant and reproducible primary outcomes, long-term follow-up, limited use of placebo and avoiding spin strategies.

## Introduction

Chronic urticaria, idiopathic or autoimmune, is a common disease affecting 0.5% to 1% of individuals (lifetime prevalence). According to various recommendations, the diagnosis of chronic urticaria is clinical. It is characterized by erythematous daily or almost daily itchy-wheals or hives lasting more than 6 weeks. Up to 40% of patients with urticaria for more than 6 months still have urticaria 10 years later and 20% have it 20 years later. Management of the disease still remains unclear despite multiple trials. Second-generation H1-antihistamines are recommended as first-line therapy; the choice and doses of antihistamines and associated drugs are not specified. Moreover, with failure of these treatments, the strategy is unclear. The place of anti-leukotrienes and immunomodulatory and immunosuppressive treatments is not defined.

Numerous guidelines [1]–[4] and expert opinions [5] have been published since 2003. However, the recommendations have heterogeneous conclusions and failed to standardize the therapeutic management. Different recommendations resulting from a sample of studies raises the question of the difficulty in interpreting results. Results from trials of good quality should be easy to interpret and should not lead to different conclusions.

Thus, we reviewed the methodological characteristics and quality of reporting of results of randomized control trials of the pharmacological treatment of autoimmune and idiopathic chronic urticaria.

## Materials and Methods

### Search Strategy, Selection of Revelant Articles

We searched MEDLINE via PubMed and EMBASE for articles published in English and French up to March 2011. The search strategy is in Appendix S1. We searched for reports of pharmacological randomized controlled trials involving patients with chronic autoimmune or idiopathic urticaria with or without angioedema, with main outcome treatment efficacy. We excluded reports of studies involving only patients with isolated angioedema or with known causes of urticaria: allergic, physical, or secondary to a general abnormality, with the exception of autoimmune urticaria. Relevant articles were identified by the title and abstract by 2 authors (EL, GL), who were blinded to each other in selecting articles. Differences were resolved by consensus. From identified reports, we selected only those published since 1996, the year of publication of the first CONSORT statement defining guidelines to improve the quality of reporting of trials [6]. We also searched for initial descriptions of published studies on the trial registration websites ClinicalTrials.gov [7] and Current Controlled Trials [8].

### Data Collection

A data collection form was complied and validated by discussion of the authors. The form was based on the model proposed by the Cochrane Collaboration, in the Cochrane Handbook for Systematic Reviews of Interventions [9], and by the CONSORT statement [6]. The form was pre-tested on 20 reports and was modified according to the results of the pre-test. One of us (EL) extracted all the data.

Data were collected on general characteristics of the studies, internal validity, studied treatments, design of the trial, outcome measures and “spin” strategy in interpreting results. Data collected on general characteristics of the studies included the name and category of the journal (dermatology, allergy and immunology journals, pharmacology and therapeutic journals, non-specialized), year of publication, funding source, and registration in an international database. Data were extracted on the definition of urticaria, etiology, duration, severity and inclusion and exclusion criteria; on internal validity, including randomization method, blinding of patients and outcome assessors, possible doubt on blinding linked to side effects, intention-to-treat analysis, number of drop-outs, and reference to the CONSORT statement; on treatments, including the name of the investigated molecules and the use of a placebo; on the design of the trial, including parallel or cross-over status, sample size calculation, number of arms, study duration, and duration of follow-up after the discontinuation of the treatment; and on outcome measures, including assessment of efficacy and whether the primary outcome was mentioned. If only one outcome was mentioned, we considered it as the primary outcome.

According to Boutron et al [10] spin can be defined as “use of specific reporting strategies, from whatever motive, to highlight that the experimental treatment is beneficial, despite a statistically nonsignificant difference for the primary outcome, or to distract the reader from statistically nonsignificant results.” Studied spin strategies included a focus on a statistically significant secondary outcome, statistically significant subgroup analyses, within-group assessment (within-group comparison, both treatments are effective, treatment administered in both groups is effective), claiming equivalence for statistically non-significant results, efficacy with no consideration of the statistically non-significant results, acknowledging statistically nonsignificant results for the primary outcome but emphasizing other statistically significant results, or acknowledging statistically nonsignificant results for the primary outcome but emphasizing the beneficial effect of treatment.

We referred to the PRISMA checklist when applicable [11]. The PRISMA checklist is provided in Appendix S2.

### Statistical Analysis

The analysis was descriptive. Data are presented as number, percentages, median and interquartile range (IQR).

## Results

### Selected Articles

**Figure 1:**
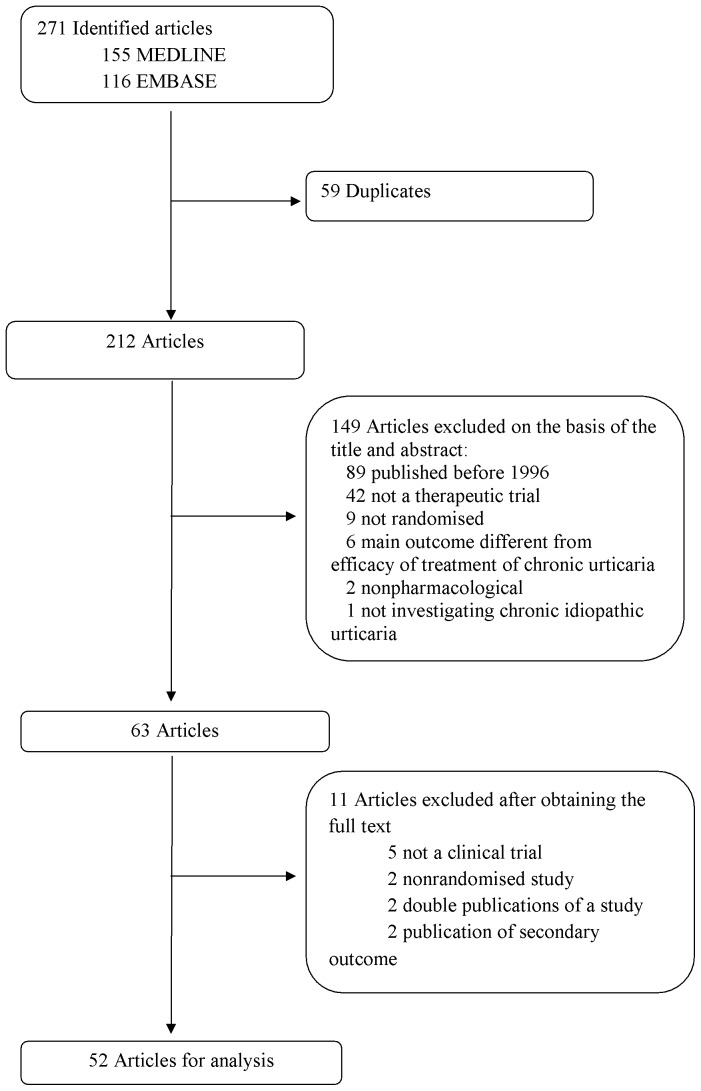
Flow diagram of selected papers.

The flowchart of selected articles is in Figure 1. The electronic search identified 271 articles: 155 from MEDLINE via PubMed and 116 from EMBASE. We retrieved the full text of the 52 articles that met our inclusion criteria. In all, 25 (48%) were published in dermatology journals, 18 (35%) in allergy and immunology journals, 6 (12%) in pharmacology and therapeutic journals, and 3 (6%) in non-specialized journals. A total of 25 reports (48%) described multicenter studies. Overall, 25 articles (48%) described private-industry funding, 3 (6%) private nonprofit funding, and 5 (10%) public funding; 21 (40%) did not mention the funding source. Six studies were registered on ClinicalTrials.gov [7]. A median of 80.5 patients (IQR 50.5–171.5 patients) were randomized per study. Table S1 shows the characteristics of the selected articles.

### Characteristics of Urticaria

The definition of urticaria was clinical in 45 reports and was not specified in 7. Three articles mentioned wheals (erythematous or not), pruritus, transient lesions, daily or almost daily disease, during more than 6 weeks and no other etiology found. Disease duration before inclusion was >6 months in 30 reports, ≤6 months in one report, and not specified in 21 reports.

The inclusion and exclusion criteria for the studies are in Table 1. The severity of urticaria required for inclusion was not specified in 26 articles (50%). For the articles that specified a severity required for inclusion, 17 (33%) described evaluation by a severity score. The other articles indicated the use of number of days without urticaria or did not specify an evaluation method.

**Table 1:** Inclusion and exclusion criteria in reports of randomized clinical trials of chronic idiopathic or autoimmune urticaria.

Inclusion and exclusion criteria	Specifications if necessary	No. of articles n = 52
Active chronic urticaria necessary for inclusion		33
Severity of urticaria needed for inclusion (severalpossibilities for one study)		
	Mild	1
	Moderate	18
	Severe	22
	Not stated	26
Autologous serum skin test realized	Total	13
	Positive results needed for inclusion	5
	Negative results needed for inclusion	2
	Results not taken into account for inclusion	6
Stopping previous treatment needed for inclusion		28
Failure of previous treatment needed for inclusion		7
Previous treatment needed for inclusion		
	Antihistamines	14
	Steroids	2
	Immunosuppressors	1
Physical urticaria excluded		33

### Internal Validity

The randomization method was specified in 16 articles (31%) (Table 2). Patients were reported as blinded to treatment in 42 (81%) and the outcome assessor was blinded in 37 (71%). Among these 37 reports, for 5, blinding was doubtful because of possible clinical or biological side effects associated with the studied treatment. Two reports described first-generation antihistamines and one benzodiazepine, which can induce sedation. This possible bias was not reported in the discussion of the articles. Two articles about cyclosporine did not specify how blinding of the outcome assessor was maintained despite clinical and biological side effects. In 4 articles (8%), blinding was clear because the outcome assessor was blinded, the treatment was not recognizable (identical or double-dummy) or had no major secondary effects and the computed randomization was centralized [12]–[15].

**Table 2:** Internal validity of articles.

Criteria of internal validity	Specifications if necessary	No. of articles n = 52
Randomization method specified	Total	16
	Computer-generated	12
	Author method	4
Location of randomization specified	Total	8
	Central randomization	6
	Local randomization	2
Patient blinding		
	Blinded	42
	Not blinded	7
	Blinding status not stated	3
	Method of blinding stated	19
	Use of similar treatments	9
	Double dummy (double placebo)	5
Outcome assessors blinding		
	Blinded	37
	Not blinded	10
	Blinding status not stated	5
	Method of blinding stated	11
Intention-to-treat analysis		
	Intention-to-treat analysis declared	22
	Declared as no intention-to-treat analysis	7
	Not stated	23
	Actually studied data for all randomized patients	7
Drop-out reported		39

In all, 22 articles (42%) described an intention-to-treat analysis and 18 (35%) gave a definition of this analysis. Among the 18 articles, 2 described real intention-to-treat analysis and 16 a modified intention-to-treat analysis. Among the 16 articles, 10 included patients who had at least one evaluation, 4 patients who received the treatment at least once and 2 patients who presented urticaria during an inclusion phase. In total, 7 articles (13%) indicated that data for all randomized patients were analyzed. A total of 39 articles (75%) described drop-outs. Seven articles did not specify the final number of participants. The median percentage of drop-outs per study was 12%, with a maximum of 52% [16].

No article referred to or referenced the CONSORT statement.

### Studied Treatments

Thirty-one different molecules were investigated. The studies investigated 13 different second-generation H1-antihistamines at least once in at least one arm of one trial. A second-generation H1-antihistamine was investigated at least in one arm in 48 articles (92%). In total, 22 articles (42%) described the comparison of H1-antihistamines; 9 described treatments other than antihistamines, antileukotrienes, levothyroxine or cyclosporine. These treatments were autologous whole blood injection, benzodiazepine, dapsone, dipyridamole, hydroxychloroquine, levamisole, stanozolol, theophylline, and total glucoside peony capsules.

A total of 32 articles (61%) described use of a placebo as a comparator, which was the only comparator in 13 (25%) (Table 3); 17 articles (33%) described comparing an antihistamine to another treatment.

**Table 3:** Studied treatments and comparators.

Treatment	Comparator(s)	No. of articles (n = 52)
**H1-antihistamines**	H1-antihistamines ± placebo	22
**H1-antihistamines**	Placebo	6
**Antileukotrienes**	H1-antihistamines and/or placebo	8
**Levothyrox**	H1-antihistamines	2
**Cyclosporine**	H1-antihistamines (3) or prednisone (1) or cyclosporine (1)	5
**Other treatments**		9

### Design of the Studies

A parallel design was used in 49 trials and a cross-over design in 3 (one started in parallel and ending in cross-over); 12 trials used 3 or 4 arms. The study duration was <8 weeks in 39 articles (75%); 37 (71%) described no follow-up after the discontinuation of treatment (Table 4). The calculation of the needed sample size was reported in 13 articles (25%) and was described as achieved in 10.

**Table 4:** Durations of treatment and follow-up after discontinuation of treatment.

Duration (treatment or follow-up)	Treatment duration No. of articles	Follow-up duration No. of articles
0	0	37
<2 weeks	0	3
2–4 weeks	5	1
4–8 weeks	34	4
8–12 weeks	3	2
≥12 weeks	9	4
Not stated	2	1

One article compared 2 different durations (cyclosporine 4 weeks *vs* 12 weeks).

### Outcome Measures

A total of 33 articles (63%) specified the primary outcome and 19 (37%) did not. In 32, the primary outcome was a score or a scale. In one article [17] the primary outcome was complete clinical remission, defined as remission for 3 days, then the patient left to pursue studies, with no follow-up. If only one outcome was mentioned, we considered it as the primary outcome: this was the case for 9 of 33 articles with a primary outcome specified.

Of the 33 articles that specified a primary outcome, 25 (76%) described a statistical comparison of results of scores of urticaria, 6 used a binary analysis of scores, giving a pre-therapeutic definition of efficacy as a percentage of decrease in a score, 1 defined efficacy as a 3-day symptom-free period, and 1 gave 3 different possible results (symptom-free, partial improvement, no improvement). Seven articles (13%) described a biological assessment of treatment efficacy as a secondary outcome.

The tools used in the 52 articles for evaluating clinical efficacy were efficacy scores, quality-of-life scales, use of rescue medication, clinical complete remission and non-described scores. These criteria were evaluated by patients and/or assessors. In total, 15 different scores were used. Different items were used at least once to determine the severity of urticaria, and the efficacy of treatments were severity of pruritus, intensity of erythema, global evaluation of wheals, extension of wheals, number and size of wheals, duration of wheals, sleep disturbance, daily disturbance and number of separate episodes. The intensity of pruritus was described in 46 articles and was the most-studied item. Each item was measured in different ways: scales of 4 or 5 points or more or visual analog scales. For example, among the 4 articles with complete reporting of blinding, scores used for the evaluation of the primary outcomes differed,involving difficulties in comparing the studies.

### Spin Strategy in Interpretation of Results

We searched for a spin strategy in the 12 articles reporting a non-significant primary outcome, among the 33 studies with a primary outcome. At least one spin strategy was observed in the discussion and/or conclusions sections in 10 of the 12 articles (Table 5). For example, among the 4 articles with complete reporting of blinding, one used a spin strategy claiming equivalence for statistically non-significant results. Authors had written “No significant difference between groups was found”, “This study shows that emedastine difumarate […] is at least as effective in controlling symptoms in idiopathic chronic urticaria in Caucasian patients as loratadine” [14].

**Table 5:** “Spin” strategy in discussion and/or conclusions sections of articles. Adapted from Boutron et al [7].

Spin strategy in discussion and/or conclusion	No. of articles (n = 10^1^)
Focus on statistically significant secondary outcome	0
Focus on statistically significant subgroup analyses	0
Focus on within-group assessment (within-group comparison, both treatments are effective, treatment administrated in both groups is effective)	5
Claiming equivalence for statistically nonsignificant results	5
Claiming efficacy with no consideration of the statistically nonsignificant results	1
Acknowledge statistically nonsignificant results for the primary outcome but emphasize other statistically significant results	1
Acknowledge statistically nonsignificant results for the primary outcome but emphasize the beneficialeffect of treatment	0

1 Two articles exhibited 2 different spin strategies.

## Discussion

Chronic urticaria was well defined in this review of the study design and quality of reporting of randomized control trials investigating drug treatment of autoimmune or idiopathic chronic urticaria. Few articles mentioned all clinical criteria, but lack of 1 or 2 criteria seemed acceptable. The study duration was <8 weeks in 39 studies (75%), with no follow-up after the discontinuation of treatment in 37 (71%). The primary outcome was specified in 33 articles. It was evaluated by 15 different scores. Double blinding was not systematic and reporting of blinding was adequate in 4 articles. A spin strategy to report findings was featured in 83% of the reports with nonsignificant results for the primary outcome.

### Duration of Studies

Chronic urticaria is a disease of variable duration and can last several years [18]. Studies of the condition are often short term and without any follow-up after the end of the treatment. These short-term studies are useful to determine the efficacy of short-term treatments, but the findings cannot be extrapolated as being efficacious for long-term treatment and do not answer whether there is a loss of the efficacy with time or what happens when treatment is discontinued. Safety studied in short-term studies cannot be transposed to long-term treatment. Finally, these short-term studies do not allow for establishing therapeutic strategies. The objective of the studies should be specified: to treat disease outbreak or for long-term treatment. Long-term studies are needed to establish recommendations for long-term treatment, not just outbreaks.

### Outcome Measures

The primary outcome was not specified in 37% of our articles. Among studies that specified a primary outcome, 15 different scores were used. This heterogeneity induces difficulties in comparing the different treatments and thus developing therapeutic strategies. The primary outcome was expressed as a statistically significant decrease in a score in 76% of articles. The clinical relevance of a significant decrease in a score is highly questionable; indeed a significant change in a score is not synonymous with significant clinical improvement. Scores are useful to evaluate the efficacy of treatments and to compare results of studies. Standardized, reproducible, and well-evaluated scores such as the urticaria activity score (UAS) [19] or quality-of-life scores such as the Dermatology Life Quality Index (DLQI) should be prefered to a “homemade” score. For clinically relevant scores, the objective should be a percentage decrease in score, such as 75% or even 90%. Scores should be systematically completed by study of complete clinical remission as a primary or secondary outcome. However, above all, satisfaction of the patient should be considered. Studies have shown a lack of patient-important outcome in studies of other diseases such as diabetes and cardiovascular risk [20], [21]. Use of composite scores should be reserved for secondary outcomes.

### Placebo

Placebo was the only comparator in 25% of the trials. Placebo is useful for the first study of a treatment to evalue its efficacy. Use of a placebo as a comparator also allows for comparing results of trials. Neverthless, choice of placebo as the only comparator can be criticized and is an ethical issue in light of the existence of first-line, well-tolerated, validated therapies. The use of a placebo is acceptable in some cases, but head-to-head studies of superiority or non-inferiority are needed to establish therapeutic strategies.

### Quality of Reporting

Studies were analyzed on the basis of full-text articles; thus, we depended on the quality of the reporting. Numerous data were missing, so we could not evaluate internal validity. For example, in the description of blinding of patients and outcome assessors, except for 4 articles, the reporting did not allow for determining whether the evaluation was effectively double blinded. Evaluation of chronic urticaria is subjective, so well-done double-blind studies are essential and the articles should allow for evaluating the quality of double-blinding. Poor quality of reporting can be linked to poor quality of studies and to the limited word count set by the different journals. A recent study underlined that the poor quality of reporting does not systematically reflect the quality of the protocols [22]. This problem of quality of reporting should be improved by systematic use of the CONSORT statement. In our study, no article referenced the CONSORT statement, a validated tool, published in 1996, to improve the quality of reporting. Items that should be reported and that we found absent in some reports were the definition of primary and secondary outcomes, the description of randomization, allocation concealment, blinding, and the calculation of a sample size. Other studies have highlighted the lack of use of the CONSORT statement in dermatological trials, and previous publications have highlighted the poor quality of published reports of dermatology [23]–[26]. In 2000, Adetugbo et al. [26] pointed to the need to use the CONSORT statement to improve the quality of published trials.

### Spin Strategy in Interpretation of Results

A spin strategy to report findings was featured in 83% of the reports with non-significant results for the primary outcome. The most-frequent strategies were within-group assessment and interpretation of nonsignificant results as similar effect. These inadequate interpretations can have implications for the reader in determining therapeutic strategy. Use of a spin strategy can be explained by publication bias. Indeed, studies with non-significant results are less-often published [27]. Some authors include a spin strategy in reports for an interesting interpretation of results to facilitate the publication of negative studies. Moreover, positive studies are needed to market treatments. Journal editors and reviewers must be diligent about identifying spin strategies. Registration of studies is also needed to allow for transparency of protocols and to avoid modifications of the protocols in case of statistically nonsignificant results.

To conclude, performing good-quality studies of treatment of chronic urticaria is difficult because of the chronicity of the disease, the subjectivity of the evaluation, and the difficulty in finding good primary outcomes. Nevertheless, all the issues that we observed can induce difficulties in comparing treatments, analyzing results, transposing results to the management of chronic urticaria and thus establishing recommendations.

## Supporting Information

Table S1 **Design of the studies.**
(DOCX)

Appendix S1 **Search Strategy: Key words used for the research.**
(DOCX)

Appendix S2 **PRISMA 2009 Checklist.**
(DOC)
