# Supplementary material for: Study Design and Quality of Reporting of Randomized Controlled Trials of Chronic Idiopathic or Autoimmune Urticaria: Review
Source: PLoS One. 2013 Aug 5;8(8):e70717. doi: 10.1371/journal.pone.0070717 (PMC3733774; doi:10.1371/journal.pone.0070717)
Supplement: Table S1 — Design of the studies. (DOCX) [file pone.0070717.s001.docx]

Table S1 : Design of the studies.

| Study | Population: | Treatments: name (duration of the study) | Study design | Primary outcome | Patient blinded | Outcome assessor blinded | Follow-up after discontinuation of treatment |
| --- | --- | --- | --- | --- | --- | --- | --- |
| Abu Shareeah AM 1998 | 29 | Cetirizine *vs* Mequitazine *vs* Placebo (3 weeks) | Parallel | Not specified | Yes | Yes | No |
| Anuradha P 2010 | 60 | Loratadine *vs* Levocetirizine (4 weeks) | Parallel | Symptom score | Yes | Yes | No |
| Bagenstose SE 2004 | 95 | Zafirlukast + Cetirizine *vs* Cetirizine + Placebo (3 weeks) | Parallel | Not specified | Yes | Yes | No |
| Baskan EB 2004 | 20 | Cyclosporine + Cetirizine *vs* Cyclosporine + Cetirizine (4 or 12 weeks) | Parallel | Symptom score | No | No | Until relapse |
| Breneman DL 1996 | 188 | Cetirizine *vs* Hydroxyzine *vs*  Placebo (4 weeks) | Parallel | Not specified | Yes | Yes | No |
| Brostoff J 1996 | 56 | Mizolastine *vs* Placebo (4 weeks) | Parallel | Symptom score | Yes | Yes | No |
| Camarasa JM 2001 | 55 | Azelastine *vs* Ebastine *vs* Placebo (3 weeks) | Parallel | Symptom score | Yes | Yes | No |
| Di Gioacchino M 2003 | 40 | Cyclosporine *vs* Cetirizine (8 weeks) | Parallel | Not specified | Yes | Yes | 9 months |
| Di Lorenzo G 2004 | 160 | Desloratadine + Placebo *vs* Desloratadine + Montelukast *vs* Montelukast + Placebo *vs* Placebo + Placebo (6 weeks) | Parallel | Symptom score | Yes | Yes | 2 weeks |
| Dubertret L 1999 | 247 | Mizolastine *vs* Loratadine *vs* Placebo (4 weeks) | Parallel | Symptom score | Yes | Yes | No |
| Dubertret L 2007 | 283 | Rupatadine *vs* Rupatadine *vs* Rupatadine*vs* Placebo (4 weeks) | Parallel | Symptom score | Yes | Yes | No |
| Duenas Leita 2009 | 12 | Alprazolam *vs* Rupatadine (Not specified) | Parallel | Not specified | Yes | Yes | No |
| Engin B 2008 | 68 | Dapsone + Desloratadine  *vs* Desloratadine (3 months) | Parallel | Symptom score | No | No | 3 months |
| Erbagci Z 2002 | 30 | Montelukast *vs* Placebo (6 weeks) | Cross-over | Not specified | Yes | No | No |
| Finn AF 1999 | 476 | Fexofenadine *vs* Placebo (4 weeks) | Parallel | Symptom score | Yes | Yes | No |
| Gimenez-Arnau A 2007 | 334 | Rupatadine *vs* Rupatadine *vs* Placebo (4 weeks) | Parallel | Symptom score | Yes | Yes | No |
| Godse KV 2006 | 20 | Montelukast *vs* Cetirizine (2 weeks) | Parallel | Symptom score | Yes | Yes | No |
| Grattan CE 2000 | 30 | Cyclosporine + Cetirizine *vs* Cetirizine + Placebo (4 weeks) | Parallel | Symptom score | Yes | Yes | 20 weeks or until clinical relapse |
| Grob JJ 2009 | 106 | Desloratadine + Placebo *vs* Desloratadine + Placebo (8 weeks) | Parallel | Quality of life score | Yes | Yes | 2 months |
| Handa S 2004 | 116 | Cetirizine *vs* Fexofenadine (4 weeks) | Parallel | Symptom score | Yes | Yes | No |
| Hong JB 2010 | 64 | Desloratadine *vs* Levocetirizine (6 weeks) | Parallel | Not specified | Yes | Yes | No |
| Kalis B 1996 | 211 | Ebastine *vs* Terfenadine *vs* Placebo (3 months) | Parallel | Not specified | Yes | Yes | No |
| Kalogeromitros D 2006 | 134 | Theophylline + Cetirizine *vs* Cetirizine + Placebo (6 months) | Parallel | Not specified | Yes | Yes | No |
| Kameyoshi Y 2007 | 21 | Cetirizine *vs* Cetirizine (2 to 4 weeks) | Parallel | Not specified | No | No | No |
| Kaplan AP 2005 | 259 | Fexofenadine *vs* Placebo (4 weeks) | Parallel | Symptom score | Yes | Yes | No |
| Kapp A 2006 | 166 | Levocetirizine *vs* Placebo (4 weeks) | Parallel | Symptom score | Yes | Yes | 1 week |
| Karaayvaz M 2002 | 60 | Ketotifen *vs* Levothyroxinie (6 weeks) | Parallel | Not specified | No | No | At least 6 months |
| Khalaf AT 2008 | 64 | Dipyridamole + Desloratadine *vs* Desloratadine + Placebo (4 weeks) | Parallel | Not specified | Yes | Yes | 4 weeks |
| Kiyici S 2010 | 15 | Levothyroxine + Desloratadine *vs* Desloratadine (12 weeks) | Parallel | Symptom score | No | No | No |
| La Rosa M 2001 | 62 | Oxatomide + Placebo *vs* Cetirizine + Placebo (4 weeks) | Parallel | Not specified | Yes | Yes | No |
| Leynadier F 2000 | 61 | Mizolastine *vs* Loratadine (4 weeks) | Parallel | Symptom score | Yes | Yes | No |
| Long JW 2010 | 120 | Total glucosides of paeony capsule + Cetirizine *vs* Cetirizine (4 weeks) | Parallel | Not specified | No | No | 1 month |
| Loria MP 2001 | 20 | Cyclosporine *vs* Prednisone (8 weeks) | Parallel | Symptom score | No | No | Not specified |
| Monroe E 2003 | 226 | Desloratadine *vs* Placebo (6 weeks) | Parallel | Symptom score | Yes | Yes | No |
| Nelson HS 2000 | 468 | Fexofenadine *vs* Placebo (4 weeks) | Parallel | Symptom score | Yes | Yes | No |
| Nettis E 2001 | 27 | Montelukast *vs* Fexofenadine (30 days) | Parallel | Symptom score | Yes | Yes | No |
| Nettis E 2004 | 81 | Montelukast + Desloratadine *vs* Desloratadine + Placebo *vs* Placebo (6 weeks) | Parallel | Not specified | Yes | Yes | 1 week |
| Nettis E 2006 | 106 | Levocetirizine *vs* Placebo (6 weeks) | Parallel | Not specified | Yes | Yes | 1 week |
| Ortonne JP 2007 | 142 | Desloratadine *vs* placebo (6 weeks) | Parallel | Symptom score | Yes | Yes | No |
| Parsad D 2001 | 58 | Stanozolol + Cetirizine *vs* Cetirizine + Placebo (12 weeks) | Parallel | Symptom score | Yes | Yes | No |
| Patel P 1998 | 46 | Loratadine *vs* Cetirizine (2 weeks) | Parallel | Symptom score | Yes | Yes | No |
| Paul E 1998 | 222 | Fexofenadine *vs* Placebo (6 weeks) | Parallel | Symptom score | Yes | Yes | No |
| Pons Guiraud A 2006 | 192 | Emedastine difumarate *vs* Loratadine (4 weeks) | Parallel | Symptom score | Yes | Yes | No |
| Potter PC 2009 | 886 | Desloratadine *vs* Levocetirizine (4 weeks) | Parallel | Symptom score | Yes | Yes | No |
| Reeves GE 2004 | 21 | Hydroxychloroquine *vs* Placebo (12 weeks) | Parallel | Not specified | Yes | Yes | No |
| Reimers A 2002 | 52 | Zafirlukast *vs* Placebo (6 weeks) | Cross-over | Not specified | Yes | Yes | No |
| Staevska M 2010 | 80 | Levocetirizine *vs* Desloratadine (4 weeks) | Parallel and cross-over | Symptom free | Yes | Yes | No |
| Staubach P 2006 | 56 | Autologus whole blood injection *vs* Placebo (8 weeks) | Parallel | Not specified | Yes | No | 4 weeks |
| Vena GA 2006 | 99 | Cyclosporine + Cetirizine *vs* Cyclosporine + Cetirizine + Placebo *vs* cetirizine + Placebo (16 weeks) | Parallel | Symptom score | Yes | Yes | 8 weeks |
| Wan KS 2009 | 120 | Hydroxyzine + Cetirizine *vs* Hydroxyzine + Famotidine *vs* Hydroxyzine + Montelukast *vs* Placebo (4 weeks) | Parallel | Symptom score | Yes | No | No |
| Zhang H 2010 | 132 | Levamisole + Levocetirizine *vs* Levocetirizine + Placebo (6 weeks) | Parallel | Symptom score | Yes | Yes | No |
| Zuberbier T 2010 | 522 | Bilastine *vs* Levocetirizine (4 weeks) | Parallel | Symptom score | Yes | Yes | No |
