# Supplementary material for: Study Design and Quality of Reporting of Randomized Controlled Trials of Chronic Idiopathic or Autoimmune Urticaria: Review
Source: PLoS One. 2013 Aug 5;8(8):e70717. doi: 10.1371/journal.pone.0070717 (PMC3733774; doi:10.1371/journal.pone.0070717)
Supplement: Appendix S1 — Search Strategy: Key words used for the research. (DOCX) [file pone.0070717.s002.docx]

**Appendix S1: Key words used for the research**

**MEDLINE**

**Chronic urticaria and antihistamines, controlled trials:**

("Urticaria"[Mesh:noexp] AND "Chronic Disease"[Mesh]) OR chronic idiopathic urticaria[Title] OR chronic autoimmune urticaria[Title]

AND

("Histamine Antagonists"[Mesh:noexp] OR "Histamine H1 Antagonists"[Mesh] OR "Histamine H1 Antagonists, Non-Sedating"[Mesh] OR "Histamine H2 Antagonists"[Mesh])

AND

("Controlled Clinical Trial"[Publication Type] OR "Randomized Controlled Trial"[Publication Type] OR "Single-Blind Method"[MeSH] OR "Double-Blind Method"[MeSH] OR "Random Allocation"[MeSH] OR "Controlled Clinical Trials as topic"[MeSH] OR "Randomized Controlled Trials as topic"[MeSH] OR "Comparative Study"[Publication Type])

**Chronic urticaria and other treatments, controlled trials:**

("Urticaria/drug therapy"[Mesh:noexp] AND "Chronic Disease"[Mesh]) OR "Urticaria"[Mesh:noexp] AND "Chronic Disease"[Mesh] OR chronic idiopathic urticaria[Title] OR chronic autoimmune urticaria[Title]

AND

("Anti-Allergic Agents"[Mesh] OR "Antibodies, Monoclonal"[Mesh:noexp] OR "Immunologic Factors"[Mesh:noexp] OR "Immunoglobulins, Intravenous"[Mesh] OR "Receptors, IgE"[Mesh] OR "Sirolimus"[Mesh] OR "Platelet Activating Factor"[Mesh] OR "Adrenal Cortex Hormones"[Mesh:noexp] OR "Steroids/therapeutic use"[Mesh] OR "Glucocorticoids/therapeutic use"[Mesh] OR "Prednisone"[Mesh] OR "Tranexamic Acid"[Mesh] OR "Alprazolam"[Mesh] OR "Cyclosporine"[Mesh] OR "Methotrexate"[Mesh] OR "Warfarin"[Mesh] OR "Phototherapy"[Mesh:noexp] OR "Ultraviolet Therapy"[Mesh:noexp] OR "PUVA Therapy"[Mesh:noexp] OR "Levamisole"[Mesh] OR "Immunoglobulins"[Mesh:noexp] OR "Tacrolimus"[Mesh] OR "Hydroxychloroquine"[Mesh] OR "Indomethacin"[Mesh:noexp] OR "Leukotriene Antagonists"[Mesh] OR "Dipyridamole"[Mesh] OR "mycophenolate mofetil"[Substance] OR "Immunosuppressive Agents"[Mesh:noexp] OR "Calcium Channel Blockers"[Mesh] OR "Antidepressive Agents, Tricyclic"[Mesh] OR "Colchicine"[Mesh:noexp] OR "Interferon-gamma"[Mesh:noexp] OR "Blood Transfusion, Autologous"[Mesh] OR "Acedapsone"[Mesh] OR "Dapsone"[Mesh] OR "Sulfasalazine"[Mesh] OR "Cyclophosphamide"[Mesh:noexp] OR "Tumor Necrosis Factor-alpha/antagonists and inhibitors"[Mesh]

AND

Humans[Mesh] AND English[lang] OR French[lang]

AND

("Controlled Clinical Trial"[Publication Type] OR "Randomized Controlled Trial"[Publication Type] OR "Single-Blind Method"[MeSH] OR "Double-Blind Method"[MeSH] OR "Random Allocation"[MeSH] OR "Controlled Clinical Trials as topic"[MeSH] OR "Randomized Controlled Trials as topic"[MeSH] OR "Comparative Study"[Publication Type])

**EMBASE**

**Chronic urticaria and antihistamines, controlled trials:**

'chronic urticaria'/de OR 'chronic urticaria':ti

AND

'antihistaminic agent'/de OR 'histamine h1 receptor antagonist'/exp OR 'histamine h2 receptor antagonist'/exp

AND

randomized controlled trial'/de OR 'controlled clinical trial'/de OR 'double blind procedure'/de OR 'crossover procedure'/de OR 'single blind procedure'/de

**Chronic urticaria and other treatments, controlled trials:**

'chronic urticaria'/de OR 'chronic urticaria':ti

AND

'monoclonal antibody'/exp OR 'antiallergic agent'/exp OR 'immunologic factor'/de OR 'immunoglobulin'/de OR 'immunoglobulin E receptor'/de OR 'rapamycin'/de OR 'thrombocyte activating factor'/de OR 'thrombocyte activating factor'/de OR 'corticosteroid'/de OR 'glucocorticoid'/de OR 'prednisone'/de OR 'tranexamic acid'/de OR 'alprazolam'/de OR 'cyclosporin'/de OR 'methotrexate'/de OR 'warfarin'/de OR 'phototherapy'/de OR 'PUVA'/de OR 'levamisole'/de OR 'tacrolimus'/de OR 'salazosulfapyridine'/de OR 'dapsone'/de OR 'acedapsone'/de OR 'colchicine'/de OR 'calcium channel blocking agent'/de OR 'immunosuppressive agent'/de OR 'dipyridamole'/de OR 'indometacin'/de OR 'leukotriene receptor blocking agent'/de OR 'hydroxychloroquine'/de OR 'mycophenolic acid 2 morpholinoethyl ester'/de OR 'tricyclic antidepressant agent'/de OR 'blood autotransfusion'/de OR 'cyclophosphamide'/de OR 'tumor necrosis factor alpha inhibitor'/de OR 'gamma interferon'/de OR 'gamma interferon'/de

AND

randomized controlled trial'/de OR 'controlled clinical trial'/de OR 'double blind procedure'/de OR 'crossover procedure'/de OR 'single blind procedure'/de
